# The War Pinch on the Huns

**Published:** 1915-08-28

**Authors:** 


### The War Pinch on the Huns.

A correspondent of the *Times* gives an interesting account of various methods which are being adopted in Germany to increase the available forms of food. The popularisation of food substitutes, he says, has been officially encouraged for a good many years, and coffee, yeast, eggs, butter, and olive oil are prominent on the list. A frequent practice among the poorer people—and not altogether disdained, at least in secret, by the middle class—is to collect acorns and roast them for admixture with ground coffee; and an extension of this and allied customs has meant that to-day the price of coffee is only some 8 per cent. higher than the prices current prior to the war. The war bread, of which so much has been heard, is described as neither attractive nor appetising, but has improved since the bakers have acquired skill in its preparation. Italian intervention has meant the reduction of the supplies of olive oil, and various sources of a new supply of oil are being investigated, one of the latest, it is said, being cherry-stones. A decidedly less attractive scheme is a plan for dealing with meat which might reasonably be condemned as unfit for human consumption. This, it seems, is in operation in Germany as a normal and usual procedure. A state butcher's shop is attached to the municipal abattoirs in the large cities, and here tainted meat, or the meat of animals suffering from localised disease, is subjected to certain specialised processes so as to remove its dangerous qualities. Commonly purchased by the poorest class, the present emergency compels its employment on a wider scale. In various other directions experts are at work in the adaptation of old substitutes or in the search for new ones. It is by methods having this aim that, according to the *Times* correspondent, Germany is managing to feed herself and to avoid the extreme prices which otherwise would certainly have prevailed. A point of special interest at the moment is the doubt thrown on the claim to have secured a process by which wood fibre may be so purified as to make it available as a substitute for cotton in the manufacture of explosives. This claim, it is suggested, is a piece of "bluff," intended to discourage the Allied Governments from declaring cotton contraband; if this were the object, the latest news on the matter shows that it has not succeeded.

The prospect for Germany is not one to maintain cheerfulness amongst its population, seeing that these various expedients have become necessary before one yard of the Fatherland has been invaded. When Germany is completely isolated from the outside world, and when the Allies seriously get to work by invading Germany in sober earnest, the outlook for non-combatants throughout the Fatherland, both in regard to food and generally, is by no means a happy one. This end is not yet.

